# Comparison between Computed Tomography and Ultrasonography in Detecting Foreign Bodies Regarding Their Composition and Depth: An *In Vitro* Study

**Published:** 2016-09

**Authors:** Abdolaziz Haghnegahdar, Alireza Shakibafard, Negar Khosravifard

**Affiliations:** 1 Dept. of Oral and Maxillofacial Radiology, School of Dentistry, Shiraz University of Medical Sciences, Shiraz, Iran.; 2 Specialist in Radiology and Sonography, TABA Medical Imaging Center, Shiraz, Iran.; 3 Postgraduate Student in Oral and Maxillofacial Radiology, Dept. of Oral and Maxillofacial Radiology, School of Dentistry, Shiraz University of Medical Sciences, Shiraz, Iran.

**Keywords:** Foreign body, Computed tomography, Ultrasonography

## Abstract

**Statement of the Problem:**

Impaction of foreign bodies in the soft tissues is a sequela of traumatic and penetrating injuries. Such foreign bodies should be removed due to the complications they cause. Patient’s history, clinical evaluation and imaging examinations aid in the proper detection and localization of the foreign bodies.

**Purpose:**

The aim of the present study was to compare the sensitivity of computed tomography (CT) and ultrasonography for detecting foreign bodies in *in-vitro* models simulating facial soft tissues.

**Materials and Method:**

Fifty foreign particles with five different compositions including wood, glass, metal, plastic, and stone were embedded in five calf tongues at 1, 2, 3, 4 and 5 cm depths. CT and ultrasonography were compared regarding their capability of detecting and localizing the foreign bodies.

**Results:**

Wood and plastic foreign bodies were demonstrated more clearly on ultrasonography images. High density materials such as metal, stone, and glass were detected with almost the same accuracy on CT and ultrasonography examinations. Visibility of the foreign bodies deteriorated on ultrasonography images as their depth increased; however, CT appearances of the foreign particles were not influenced by their depths.

**Conclusion:**

Ultrasonography is an appropriate technique for detection of foreign bodies especially the ones with low density. Therefore, it seems logical to perform ultrasonography in combination with CT in cases with the suspicion of foreign body impaction.

## Introduction

Foreign bodies are objects originating outside the body. Most often they are retained in body tissues due to car accidents, explosion ns and gunshot injuries and further complicate the patients’ situation.[1-2] Oral and maxillofacial surgeons frequently come upon foreign bodies. Factors such as size, difficult access, and close anatomic relationship of the foreign body to vital structures can present diagnostic challenges to the surgeons.[3-4]

Foreign bodies are either inert or irritating. The irritating ones cause inflammation, infection, abscess formation, pain and scarring.[5] Furthermore, they can obstruct pathways either by their size or by the scarring they cause. Besides, some foreign bodies are toxic.[6] Location and composition of the foreign bodies can vary considerably based on their route of entrance into the body tissues.[1] Regarding their composition, the most frequent foreign bodies are wood, glass and metal.[2, 7]

In addition, there is increasing number of reports related to stone foreign bodies in maxillofacial surgery.[8]

Removal of foreign bodies can be delayed in approximately one third of all cases due to initial radiographic missing or misdiagnosing.[9] Therefore, selecting an appropriate imaging technique is crucial for proper recognition of foreign bodies. Several imaging modalities including conventional plain radiography, computed tomography (CT) scans, ultrasonography and MRI have been evaluated *in vivo* and *in vitro* for locating foreign bodies.[10-14] MRI seems to be the least suitable method as particles with metallic contents at times could have hazardous movements due to the strong magnetic field.[10, 15] Furthermore, foreign bodies of almost all compositions are seen as low signal areas on MR images; thus, appearing indistinguishable from structures such as calcifications and scar tissues.[2] CT and in particular ultrasonography have been proved to be appropriate for foreign body detection in soft tissues.[7, 16] Ultrasonography has been shown to be an accurate method for detection and localization of radiolucent foreign bodies.[5, 16] It has been postulated that superficial foreign bodies with low density are detected more effectively by ultrasonography than CT and conventional plain radiography.[1]

Considering the high patient exposure dose in CT and the concerning that CT is hypothesized to be not as effective as ultrasonography in detecting low-density foreign bodies, the present study was conducted to compare CT and ultrasonography in detecting wood, plastic, glass, stone and metal foreign bodies. Moreover, the effect of impaction depth of the foreign bodies on their visibility was also evaluated. 

## Materials and Method

**Figure 1:**
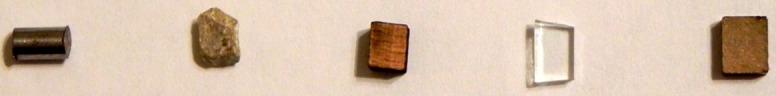
Metal, stone, wood, glass, and plastic were the foreign bodies used in this experiment.

Fifty particles with five different compositions including wood, glass, metal (stainless steel), plastic (acrylic sheet), and stone were used as foreign bodies for this *in vitro* study (Figure 1).

The rationale for selection of the mentioned materials was that they are the most frequent foreign bodies retained in human tissues. All particles had volumes in the range of 40-45 mm^3^. Initially, radiodensities of the particles were determined in Hounsfield Units (HU) by means of a CT scanner (GE VCT; General Electric, United States). Table 1 shows the HUs of the substances.

**Table 1:** Radiodensities of the investigated foreign particles in Hounsfield units (HUs)

**Foreign body**	**Range of HUs**	**Mean HU**
Wood	-239 - 0	-220
Glass	1607 - 1952	1800
Plastic	112 - 133	124
Metal	3071 - 3071	3071
Stone	2791 - 3071	3012

Five fresh calf tongues were used as representatives of maxillofacial soft tissues in the present study. All examinations were performed one day after the calves’ death. We intended to evaluate the visibility of particles at 1, 2, 3, 4 and 5 cm depths. In each of the tongues, ten particles were placed in two separate rows. Each row contained five objects with different compositions. The two rows in each tongue were created at different depths. The incisions were made by using a scalpel and sutured in order to fix the particles and cover their surfaces with soft tissue.

CT and ultrasonography were the imaging modalities compared for their capability of detecting foreign bodies. In order to enhance the reliability of the results of examinations, the visibility of each material in each depth was evaluated twice in two separate specimens. 

CT scans were obtained from GE VCT 64 slice CT scanner device (General Electric; United States) with 120 KV, 130 mA, 1.7 s scan time, 1 mm slice thickness and pitch of 1. Ultrasound scanning was performed by means of a linear transducer (Medison Accuvix V10; Samsung Medison, Korea) with frequencies in the range of 8-10 MHz.

The images were evaluated by a skilled radiologist with 20 years of experience. The observer was unaware of the location, composition and depth of the embedded foreign particles. A three-point scoring scale which allocated “no image” to (0) and “good image” to (3) was utilized for assessing the visibility of foreign bodies in CT and ultrasonography (Table 2).

**Table 2:** Scoring scale used for interpretation of the images

**Score**	**Visibility**	**Criteria of definition**
3	Good image	Good resolution of details, good demarcation from surroundings
2	Fair image	Insufficient resolution of details, insufficient demarcation from surroundings
1	Bad image	Details not resolved, bad demarcation from surroundings
0	No image	Invisible

All data were analyzed by using SPSS software version 17.0 (Chicago; IL, USA). Wilcoxon signed-ranks test was used for evaluating the difference in visibility scores of each material between CT and ultrasonography regardless of the depth. The relationship between particle visibility score and its depth in each imaging technique was assessed by using Spearman’s correlation coefficient. *p*< 0.05 was considered to be statistically significant.

## Results

**Wood**

**Figure 2:**
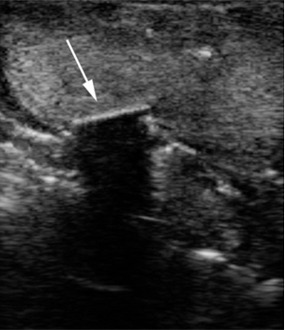
Arrow points to a wooden foreign body on the ultrasound image.

Wooden foreign bodies were well recognized by ultrasonography up to the depth of 4 cm; however, CT was unable to detect wood particles (Figure 2).

**Glass**

**Figure 3:**
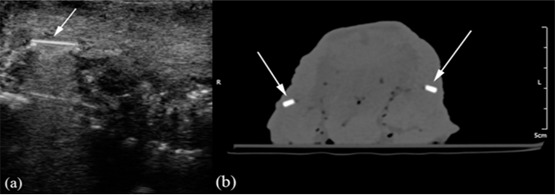
a: Arrow points to a glass foreign body on the ultrasound image. b: Arrows indicate two glass foreign bodies on the CT image.

CT depicted deeply impacted glass particles more clearly than ultrasonography; however, superficial particles were equally well demonstrated on both techniques (Figure 3a and b).

**Metal**

**Figure 4:**
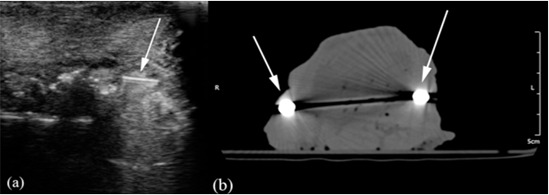
a: Arrow points to a metal foreign body on the ultrasound image. b: Arrows indicate two metal foreign bodies on the CT image (The dark streak in the middle of the image is caused by the beam hardening artifacts caused by the presence of bilateral metal particles).

Metal particles embedded at less than 4-cm depths were detected with the same accuracy in CT and ultrasonography (Figure 4a and b).

**Plastic**

**Figure 5:**
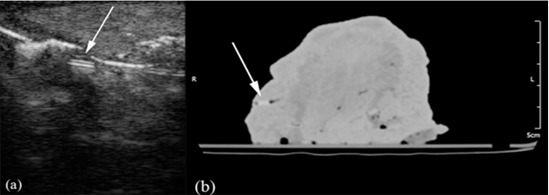
a: Arrow points to a plastic foreign body on the ultrasound image. b: Arrow indicates a plastic foreign body on the CT image.

Ultrasonography detected plastic particles more effectively than CT up to the depth of 4 cm (Figure 5a and b).

**Stone**

**Figure 6:**
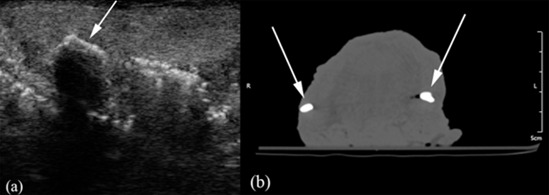
a: Arrow points to a stone foreign body on the ultrasound image. b: Arrows indicate two stone foreign bodies on the CT image.

Accuracy of CT and ultrasonography in detecting stone foreign bodies were similar at depths less than 4 cm (Figure 6a and b).

tables 3 and 4 represent the visibility scores of the specimens of the foreign bodies in CT and ultrasonography, respectively.

**Table 3:** Visibility scores of foreign bodies on CT examinations

**Foreign body**	**Depth (cm)**	**Visibility scores**
Wood	1	0
	2	0
	3	0
	4	0
	5	0
Glass	1	3
	2	3
	3	3
	4	3
	5	3
Metal	1	3
	2	3
	3	3
	4	3
	5	3
Plastic	1	1
	2	1
	3	1
	4	1
	5	1
Stone	1	3
	2	3
	3	3
	4	3
	5	3

**Table 4:** Visibility scores of foreign bodies on ultrasonography examinations

**Foreign body**	**Depth (cm)**	**Visibility scores**
Wood	1	2
	2	2
	3	2
	4	1
	5	0
Glass	1	3
	2	3
	3	2
	4	2
	5	0
Metal	1	3
	2	3
	3	3
	4	2
	5	0
Plastic	1	3
	2	2
	3	2
	4	1
	5	0
Stone	1	3
	2	3
	3	3
	4	2
	5	0

**Comparative statistical analysis of CT and ultrasonography**

Regardless of the depth of the foreign bodies, Wilcoxon signed-ranks test revealed that the visibility of wood and plastic in ultrasonography was significantly superior to CT. On the other hand, CT was more efficient in detecting glass particles compared to ultrasonography. No significant differences existed in the visibility scores of metal and stone foreign bodies between CT and ultrasonography. Table 5 demonstrates the comparison between CT and ultrasonography in detecting foreign bodies regardless of their depth.

**Table 5:** Comparison of CT and ultrasonography(US) in detecting foreign bodies regardless of depth

**Foreign body**	** P _Wilcoxon_**	**Difference between CT and US**
Wood	0.01	US significantly superior to CT
Glass	0.038	CT significantly superior to US
Metal	0.063	No significant difference
Plastic	0.038	US significantly superior to CT
Stone	0.102	No significant difference

A significant and negative correlation existed between the depth of all materials and their ultrasonographic visibility scores; nevertheless, CT appearance of the foreign bodies was uncorrelated with their depth. Table 6 presents the relationships between foreign bodies’ impaction depths and their visibility scores in ultrasonography and CT.

**Table 6:** Correlation between foreign bodies’ depths and their visibility scores

**Foreign body**	**Relationship between depth and visibility in US**		**Relationship between depth and visibility in CT**	
	r*	**P**	**r**	P
Wood	-0.926	< 0.001	N/A^†^	N/A^†^
Glass	-0.909	< 0.001	N/A^†^	N/A^†^
Metal	-0.894	< 0.001	N/A^†^	N/A^†^
Plastic	-0.930	< 0.001	N/A^†^	N/A^†^
Stone	-0.791	0.006	N/A^†^	N/A^†^

*: Spearman’s correlation coefficient,

^†^: foreign body’s visibility remained fixed at all depths on CT examinations; therefore, no correlation coefficient could be computed.

US: ultrasonography

## Discussion

Impaction of foreign bodies in the soft tissues is a sequela of traumatic and penetrating injuries.[17-18] Such foreign bodies should be removed as they can interfere with the healing process of the tissues;[7] therefore, proper detection and localization of them are imperative. The efficacy of foreign body detection depends on its composition, size and location.[17] Definitely, the employed imaging method is also of great importance.

Investigations regarding foreign body detection usually use *in vitro* models.[7, 14, 19-20] because in studies performed *in vivo*, the examiners often have knowledge of other imaging results and there is inadequate control over the size, composition and location of the foreign bodies.[21] There are also some problems with *in vitro* models including lack of the ability to in duce inflammatory reactions and other body responses around the foreign bodies.[22]

In the present study we used calf tongue due to its resemblance to the soft tissues of the face.[7] The materials evaluated for their detectability were wood, glass, metal, plastic and stone as they are frequently encountered in cases of foreign body impaction.[2, 7] Initially, Hounsfield Units of the materials were assessed by means of a CT scanner as a benchmark for their densities.

Previous studies have assessed the visibility of foreign particles having volumes of about 100 mm^3^.[1]Detection of foreign bodies becomes more complicated as they decrease in size;[2] therefore, in this study, smaller particles with volumes in the range of 40-45 mm^3^ were evaluated for their visibility. Bearing in mind the importance of how deep a foreign object is located within the tissues particles were embedded at 1, 2, 3, 4 and 5 cm depths in this investigation. Thus, the impact of foreign body depth on its visibility was also evaluated.

Various imaging methods have been employed for detecting and localizing foreign bodies. Conventional plain radiographs are incapable of demonstrating radiolucent foreign bodies such as wood.[21, 23] Xeroradiography also is inadequate for detection of wooden foreign bodies with a false negative rate of 80%; thus, it has no benefit over plain radiography.[24] MRI seems unsuitable for foreign body detection as it is difficult to distinguish low signal intensity objects from the adjacent low signal structures such as tendons, scar tissues, and calcifications in this imaging modality.[25] Moreover, displacement of the metallic foreign bodies due to the magnetic field could be life threatening in case these foreign bodies are situated adjacent to vital structures.[2] CT allows for the precise localization of the foreign bodies as a prerequisite for surgical removal;[12, 26-27] however, some studies reported that low-density foreign bodies are not detected effectively by CT.[1, 7] Ultrasonography has emerged as an appropriate imaging modality due to its widespread availability, relatively low cost, and the reported 95% sensitivity for foreign body detection.[20, 28-29]As they appear to be the most applicable imaging modalities, CT and ultrasonography were used for foreign body detection in the present investigation.

The three-point scoring scale used for evaluating the visibility of foreign bodies in the present study was a modification of the grading scale employed by Aras *et al.*, Javadrashid *et al.*, and Eggers *et al*.[1, 2, 17] which anchored “no image” to (0) and “excellent image” to (4). Our scoring scale allocated “no image” to (0), “bad image” to (1), “fair image” to (2) and “good image” to (3). In our scale, resolution of the foreign body image and its demarcation with the surrounding tissues were considered as the assessment criteria. Moreover, the (4) score was omitted compared to the previous studies as the distinction between the “excellent image” and “good image” is fairly subjective. This is not a problem with the other scores, as clear distinction could be made among “no image”, “bad image”, “fair image” and “good image” regarding the resolution and demarcation of the foreign body image. However, “good image” and “excellent image” are so indiscriminate that such a distinction could not be performed confidently.

Stone and metal foreign bodies were detected equally well on CT and ultrasonography images; a finding in conformity with previous reports.[1, 2, 7] Stone appeared as a hyperechoic area with pronounced acoustic shadow on ultrasonographic images and as a hyperdense area on CT. The ultrasonographic appearance of metal was that of a hyperechoic area with reverberation artifacts. The mentioned artifacts not only cause no diagnostic errors but also give clue to the presence of an object within the tissues. Metal particles produced streak artifacts during CT imaging; however, these artifacts did not cause any localization errors.

Glass particles were slightly better demonstrated on CT images. CT depicted glass foreign bodies as well-demarcated hyperdense areas. On ultrasonographic images, glass particles generally appeared hyperechoic with reverberation artifacts; however, in a few instances where the mentioned artifacts were not produced by the glass particles, CT could be considered superior to ultrasonography in demonstrating these foreign bodies.  

Plastic foreign bodies were much better showed on ultrasonography compared to CT. Ultrasonography illustrated plastic particles as hyperechoic areas with slight acoustic shadowing; however, CT demonstrated plastic as an area of faint density. 

Wooden foreign bodies were well visualized on ultrasonography images, while not visible on CT examinations. A number of previous reports also came to the same conclusion regarding wooden foreign bodies.[1, 2, 7] Ultrasonographic examinations exhibited wood as a hyperechoic area with acoustic shadow, whereas CT appearance of wood was that of a hypodense lacuna. In a case reported by Adesanya and Dawkins, an intraorbital wooden foreign body mimicked air on CT.[30]

The ultrasonographic appearance of each material was strongly negatively related to its impaction depth. At the depths of 1 and 2 cm, foreign bodies were best visualized. The visibility was still acceptable at 3 cm depth; however, fair visibility of the materials was encountered at the depth of 4 cm. No foreign bodies were detectable at 5 cm depth in the frequency ranges that we used (8-10 MHz). Thus, depth of 4 cm could be considered as the threshold for ultrasonographic identification of the foreign bodies within soft tissues. Penetrability of the ultrasound waves could be enhanced by reducing their frequencies to the range of 3.5-5 MHz; nevertheless, such range of frequencies would not provide the resolution required for the detection of tiny foreign bodies.[31] Visibility of the foreign bodies was not affected by their depth on CT examinations.

Inability of ultrasonography in detecting deeply impacted foreign bodies perhaps could not be considered as a true shortcoming of the technique, as low density objects that are best portrayed on ultrasonographic  images, in fact, do not penetrate a long distance through the tissues due to their physical properties. Additionally, it has been proved by this study and previous investigations[1-2,7, 32] that low-density foreign bodies such as wood and plastic are almost invisible on techniques other than ultrasonography regardless of whether they are superficially or deeply impacted.

## Conclusion

Ultrasonography could be considered as the modality of choice for detecting and localizing impacted wooden and plastic foreign bodies. High-density objects such as metal, stone and glass are illustrated with about the same clarity in CT and ultrasonography. As the depth of a foreign body increases, its visibility on ultrasonography images degrades correspondingly; however, CT appearance of the foreign body remains unaffected. In conclusion, considering the widespread availability and non-invasive nature of ultrasonography and the fact that this technique detects low-density foreign bodies with remarkable accuracy, it is advisable to supplement CT examinations with ultrasonography for patients in whom the suspicion of foreign body impaction exists. 
